# A structural and functional magnetic resonance imaging dataset of brain tumour patients

**DOI:** 10.1038/sdata.2016.3

**Published:** 2016-02-02

**Authors:** Cyril R. Pernet, Krzysztof J. Gorgolewski, Dominic Job, David Rodriguez, Ian Whittle, Joanna Wardlaw

**Affiliations:** 1 Brain Research Imaging Centre, The University of Edinburgh, Edinburgh EH4 2XU, UK; 2 Centre for Clinical Brain Sciences, The University of Edinburgh, Edinburgh EH16 4SB, UK; 3 Department of Psychology, Stanford University, Stanford, California 94305, USA; 4 Division of Clinical Neuroscience, NHS-Lothian, Edinburgh EH4 2XU, UK

**Keywords:** Outcomes research, Neurology

## Abstract

We collected high resolution structural (T1, T2, DWI) and several functional (BOLD T2*) MRI data in 22 patients with different types of brain tumours. Functional imaging protocols included a motor task, a verb generation task, a word repetition task and resting state. Imaging data are complemented by demographics (age, sex, handedness, and pathology), behavioural results to motor and cognitive tests and direct cortical electrical stimulation data (pictures of stimulation sites with outcomes) performed during surgery. Altogether, these data are suited to test functional imaging methods for single subject analyses, in particular methods that focus on locating eloquent cortical areas, critical functional and/or structural network hubs, and predict patient status based on imaging data (presurgical mapping).

## Background & summary

Soon after its inception, **f**unctional **M**agnetic **R**esonance **I**maging (**fMRI**) was used to guide brain tumour surgery^1^, and it is nowadays used in almost all areas of neurology research, from developmental to psychiatric disorders, dementia and stroke^2^. Despite this popularity in clinical research and its promising utility for surgical planning^3^, it is not used extensively in day to day clinical practice because, among other factors, of its limited accuracy, so far, at delineating eloquent areas in single subjects (but see Gorgolewski *et al.*^4,5^). The clinical data presented here were acquired in the context of a pilot study examining the feasibility and utility of fMRI for brain tumour surgical planning, with the aim of developing and validating techniques to translate fMRI cognitive paradigms to improved clinical outcomes^6^. Importantly, these clinical data can also be compared with another freely available dataset obtained in healthy volunteers, which used the exact same MRI protocol^7^.

Collecting and analysing data from brain tumour patients is challenging because of: (i) the variety of tumour types and locations; (ii) the variety of behavioural and cognitive deficits observed pre- and post- surgery, even for patients that have the same tumour type and at similar locations; and (iii) the almost unavoidable missing data, due to prioritising (for obvious ethical reasons) patients health over research data acquisition. For these reasons there are very few other MRI clinical data available, beside the Multimodal Brain Tumour Image Segmentation Benchmark data^8^ which are useful for structural imaging method development. The data presented here are well suited to test functional imaging methods for single subjects, in particular methods that focus on locating eloquent cortical areas, critical functional and structural network hubs, and predict patient status (recovery versus additional deficits caused by surgery versus behavioural improvement) based on imaging data. They can also be used to examine integrated measurements (task based fMRI, with resting state fMRI and **D**iffusion **T**ensor **I**maging (**DTI**) network analysis) to investigate issues of brain plasticity and associated behavioural performances at the group level, therefore serving both the biomedical and data science communities.

## Methods

The study was approved by the NHS Lothian South East Scotland Research Ethics Committee (REC reference number: 05/S1104/45). Patients gave informed consent for the study, including sharing of anonymised data, for non-commercial purposes (annex 1).

### Patient recruitment

**Figure 1:**
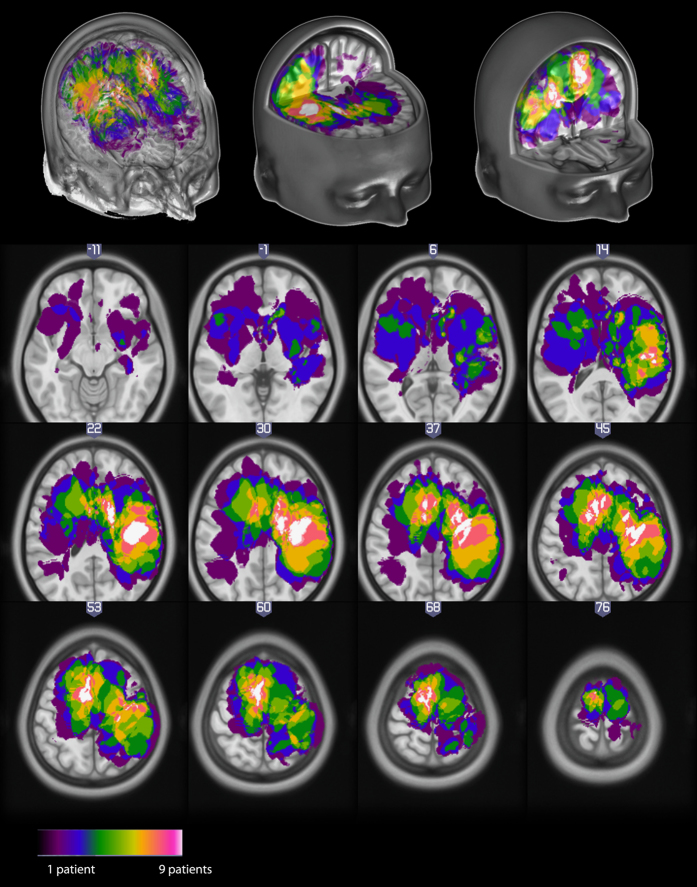
Distribution of tumours across all patients, displayed in MNI space using neurological convention.

Patients (9 Females, 13 Males, aged 25 to 75) were recruited by IW during his clinical consultations. Patients were asked to participate in the study when (i) they had a tumour located in or near a potentially eloquent area that could be mapped (motor cortex, Broca area, auditory cortex and Wernicke area) and (ii) their case posed problems in planning the surgery (e.g., deep tumour presumed to be located under/behind an eloquent area). There were no other inclusion or exclusion criteria. Most tumours were located around the pre-central region above the temporal plane (see Figure 1).

### Imaging data

**Figure 2:**
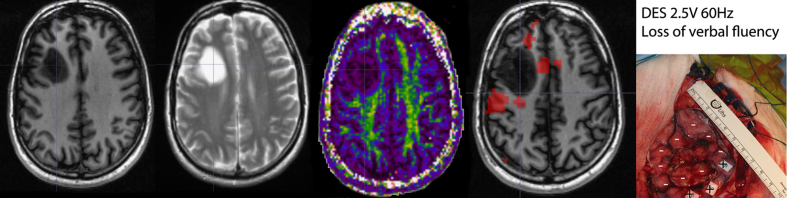
From left to right, the first 3 images show structural data: T1-weighted, T2-weighted, Diffusion weighted (Fractional Anisotropy map here) images. These data are complemented by functional BOLD imaging and Direct Electrical Stimulation.

Data were acquired on a GE Signa HDxt 1.5 Tesla scanner with an 8 channel phased-array head coil at the Brain Research Imaging Centre, University of Edinburgh, UK. *Structural Imaging Data* includes high resolution T1-weighted and T2-weighted MRI data collected along with Diffusion weighted imaging suitable for diffusion tensor imaging. *Functional Imaging Data* include up to 4 protocols per patient: motor task, verb generation task, word repetition task and resting state (an example of data is shown Figure 2, see Table 1 for details). Prior to using these protocols on a clinical population, we validated them in ten healthy controls using a test-retest design^4^ to identify tests that were reliable at the single subject level. As mentioned above, data from this study are also publicly available^7^ and can be combined with the currently presented dataset. Functional MRI stimuli were presented using the Nordic Neurolab visual and auditory system. Presentation software was used to code the different protocols. The code and stimuli are also available, along with the aforementioned test-retest dataset. Image acquisition parameters for structural and functional data are outlined in Table 2.

T1 and T2 images were defaced using SPM12, i.e., after computing the affine transform to standard space, the whole area from the forehead to the throat was removed (nullified). Tissue classes and volume estimates are also made available. For each subject, a mask of the tumour and oedema was generated semi-automatically using MRIcron 3D fill tool on the T1 and/or T2 images. This mask was then warped into standard space by estimating deformation form the T1 which allowed to create a new *a priori* tissue class. Data were then segmented using the ‘new segment’ in SPM12 using the standard priors from the MNI template, augmented by the new tumour class. Volume estimates were computed based on the segmented data and wrapping field obtained as part of the SPM segmentation.

### Direct electrical stimulation

DES during neurosurgery was performed in 17 patients. Patients were first anesthetized using propofol or fentanyl in infusion, followed by craniotomy to expose the cortex over and around the tumour using image guidance, and then wakened for DES while in the operating theatre Figure 2. When the electrical current is applied over primary sensory-motor areas, a positive outcome is expected. In the current study, DES over the different parts of the primary motor cortex was expected to induce movements in the associated boby parts. When DES is applied over secondary and associative areas, a negative outcome is expected. In the current study, stimulation of Wernicke or Broca area, a speech impairment or arrest was expected. For each patient undergoing DES procedure a digital photograph of the exposed cortex was taken (prior to the tumour resection), annotated with stimulation sites and the effect of the electrical stimulation. The presence/absence of significant fMRI activations compared to the effect of DES allowed to demonstrate here a good correspondence between techniques^6^ when using an adaptive thresholding method^4^.

### Behavioural data

Data were collected pre- and post-surgery by IW to evaluate impairments before surgery, and evaluate changes following surgery. The assessment of arm and hand function for both the dominant and non-dominant hands was performed using the nine hole peg test (9HPT), while assessment of lower limbs function was performed using a timed 10 meters walk. Five cognitive tests were also performed. The National Adult Reading Test (NART) was used to estimate premorbid intelligence levels of English-speaking patients. The Rey Adult Verbal learning test (RAVLT) was used to evaluating verbal learning and memory. The Williams delayed recall test (WDRT) was used to also provide an estimate of memory performance. The trail making test (TMT) was used to provide information about visual search speed, scanning, mental flexibility and executive functioning. Finally, the controlled oral word association test (COWAT) was used to test verbal fluency. Table 3 (available online only) details which tests were performed in which subjects pre- and post- surgery.

## Data Records

The data are available through the UK data service (http://ukdataservice.ac.uk/) under collection name: ‘A neuroimaging dataset of brain tumour patients’ (Data Citation 1).

### Demographics and behavioural data

These are considered as metadata, informing the imaging data. There is one metadata.pdf summarizing the data and a metadata.xlsx file (and the corresponding MRI_DES_data.csv and clinical_data.csv files). In this excel or csv files the age, sex, actual score values to tests and dates of these exam are provided.

### Imaging data

Data from each patient is available as a tar file with the ID provided in Table 1. Inside the archive is a folder with sub-ID which contains the data organized as zip files. Each zip file names reflects the order in which data were acquired and the sequence name. For instance, 5_finger_foot_lips.zip, 7_resting_state.zip, 8_Axial_T2.zip, 10_cor_3D_IR_PREP.zip, 10_DTI_64G_2.0-mm_isotropic.zip indicates that we started by the fMRI motor task, then resting state and moved onto structural imaging. Note that when DTI was obtained, the average diffusion coefficient, fractional anisotropy, and traceotropic maps were computed automatically by the scanner and made also available here. Finally, there is tissue_classes.zip file which contains the gray, white, csf and tumour tissue images in subject space along with a y*.nii file which corresponds t the deformation field that can be applied to transform data into MNI space). All structural and functional data are in NIfTI format (http://nifti.nimh.nih.gov/). All imaging data have been de-identified, and structural MRI data (T1, T2) were defaced.

### Direct electrical stimulation

Along with the imaging data, there is a DES.zip file that contains pictures (.jpg) taken during surgery, indicating sites of stimulation, along with a pdf file specifying additional information like e.g., left/right/front/back.

## Technical Validation

Diffusion weighted data and functional MRI protocols used in this study were validated in a set of 10 healthy participants, showing reliable patterns in a test-retest setting. For DTI, we showed that reliable single subject network metric can be obtained from the sequences used^9^. For fMRI data, we showed that tasks used have a high single subject reliability^5^ providing that an adaptive thresholding method is used to account for activation shifts in globals^4^.

## Usage Notes

The imaging data are available only after registration on the UK data service and a request is made. Although the data are de-identified and faces were removed from T1 and T2 imaging data, the detailed clinical records (age, sex, tumour, behavioural deficits, etc.,) might be enough to identify individuals directly^10^ or by using database cross-linkage^11^. Data are also available only for non-commercial use, as consented by patients. For these reasons, imaging data are protected by the UK national database user agreement.

## Additional Information

Table 3 is only available in the online version of this paper.

**How to cite this article:** Pernet, C. R. *et al.* A structural and functional magnetic resonance imaging dataset of brain tumour patients. *Sci. Data* 3:160003 doi: 10.1038/sdata.2016.3 (2016).

## Supplementary Material



## Figures and Tables

**Table 1:** Patients’ information with handedness, pathology obtained from histology (nil if not obtained), tumour location and volume, and details of the MRI sequences obtained along with DES.

**ID**	**Sub-ID**	**Handedness**	**Pathology**	**Tumour Location**	**Volume**	**Structural**	**Functional**	**DES**
693ad701-54d9-4d33-a1bb-ac047ee507d4	18638	R	Astrocytoma type II	Right primary somatosensory area	0.056	T1-T2-DTI	M	Y
7a11a476-466c-4a9e-9887-db0f80542f73	19227	R	Astrocytoma type II	Right primary somatosensory area	0.068	DTI	M-V-W	Y
9ef1729e-6db1-4c36-a77c-1ee171a4c267	18886	L	Astrocytoma type II	Right Insula	0.189	T1-T2-DTI	M-V-R	Y
c2f5ba68-d7cb-46f7-8988-397dc59da908	19567	R	Astrocytoma type II	Left Pre-Motor area	0.038	T1-T2-DTI	M-V-W	Y
cd9ff8d9-2678-41e5-8633-c3942d8069e4	18582	R	Astrocytoma type II	Wernicke area	0.021	T1-T2-DTI	M-V-W	N
be8f5123-872f-4dcb-ac96-2450e28041c1	19723	L	Astrocytoma type II	Left temporal cortex	0.091	T1-T2-DTI	MV-W-R	Y
40b10c52-6cba-45c4-af41-ed23081782b6	18428	R	Astrocytoma type III	Right Supplementary Motor Area	0.054	T1-T2-DTI	M-R	Y
03d4a4a9-e33a-4c53-9c55-b81aad73911e	19423	R	Glioblastoma Multiform	Right primary motor area	0.112	T1-T2-DTI	M-R	Y
0153c89e-aed5-48e9-a564-30fe5ceefb24	19357	R	Glioblastoma Multiform	Right primary motor area	0.099	T1-T2-DTI	M-R	N
9bba1062-9dd7-4d4a-a668-85b337fbbf64	18975	R	Glioblastoma Multiform	Left primary motor area	0.017	T1-T2-DTI	M-V-W-R	Y
458066eb-478f-4814-aab1-cad1548c320e	19015	R	Glioblastoma Multiform	Left Supplementary Motor Area	0.03	T1-T2-DTI	M-V-W	Y
d1c8edef-fafc-4e98-8f9e-67ca5823243c	18756	R	Glioblastoma Multiform	Right primary motor area	0.056	T1-T2-DTI	M-R	Y
8987684c-859f-4ea7-9cd0-4371adc4a70e	18716	R	Glioblastoma Multiform	Right primary motor area	0.096	T1-T2-DTI	M-V-W	N
95733f11-aa48-4a2b-b478-cb02f4ded3ef	19085	R	Meningioma	Right Supplementary Motor Area	0.052	T1-T2-DTI	M-R	Y
61f986b2-a38e-4ce1-8c6d-4e32d4acebba	19275	R	Meningioma	Right Pre-Motor Area	0.031	T1-T2-DTI	M	N
7b7258d1-6569-42fd-94df-a41fdf462020	19628	R	Meningioma	Left Pre-Motor area	0.164	T1-T2-DTI	M-V-W-R	N
a3f242b3-3e8c-40f7-9be0-7ff70e5d5690	18675	R	Meningioma	Left primary motor area	0.104	T1-T2-DTI	M-V-W	Y
5bcbc416-f72a-46a0-a222-fb7b45758452	19691	R	Oligodendrocytoma type II	Right Pre-Motor area	0.037	T1-T2-DTI	M-R	Y
136d3b26-a0a3-412c-99ec-ba3ece3e6177	17904	R	Oligodendrocytoma type II	Left Supplementary Motor Area	0.031	T1-T2-DTI	M-V-R	Y
5be343d8-355e-446d-88bb-7f70574a583e	19849	R	nil	Right primary motor area	0.007	T1-T2	M-R	Y
f9860d61-19c4-4698-9557-b580a57e8e8c	19398	R	Met lung CA	Right primary somatosensory area	0.064	T1-T2-DTI	M-R	Y
9517da5f-ff85-40c5-a348-1ce362a7b997	18863	L	renal cell carcinoma	Left Pre-Motor Area	0.104	T1-T2-DTI	M-V-W	Y
Sex and age are also available with the data but not presented here to avoid possible identification.								

**Table 2:** Summary of MRI sequences and parameters.

**Sequence**	**T1**	**T2**	**DWI**	**Motor task**	**Word repetition**	**Verb generation**	**Resting state**
Pulse type	3D IRP (inversion recovery prepared)	FAST spin-echo	single-shot spin-echo EPI	single-shot gradient-echo EPI			
In plane FOV				256×256 mm			
In plane matrix				256×256				128×128	64×64			
Slice thickness				1.3 mm				2 mm	4 mm			
Number of slices	156			72				30			
Acquisition order	front to back			top to bottom				interleaved			
Slice orientation	coronal			axial				axial (AC-PC)			
TR	10 s	2.5 s	16.5 s	5 s	2.5 s					
TE	4 s	102 ms	98 ms	50 ms						
Excitation flip angle	8	90	90	90						
Sparse sampling	n/a	n/a	n/a	2.5 s	n/a	n/a	n/a			
Nb of volumes in the time series	1	1	71 (7×b=0 and 64×b=1,000 s/mm2)	76	173	184	120			

**Table 3:** Details of which test scores are available pre- post- surgery for each patient (Y for yes collected, N for not collected).

**ID**	**Sub-ID**	**NART**	**RAVLT**	**Trail A**	**Trail B**	**9HPT R**	**9HPT L**	**COWAT**	**WDRT**	**10m walk**
693ad701-54d9-4d33-a1bb-ac047ee507d4	18638	Y	N	Y	Y	Y	Y	N	Y	Y
		N	N	Y	Y	Y	Y	N	Y	Y
7a11a476-466c-4a9e-9887-db0f80542f73	19227	N	N	Y	Y	N	Y	Y	Y	Y
		N	N	Y	Y	N	Y	Y	Y	Y
9ef1729e-6db1-4c36-a77c-1ee171a4c267	18886	Y	Y	Y	Y	Y	Y	Y	Y	Y
		N	N	Y	Y	Y	Y	Y	Y	Y
c2f5ba68-d7cb-46f7-8988-397dc59da908	19567	N	Y	Y	Y	Y	Y	Y	Y	N
		N	N	N	N	N	N	N	N	N
cd9ff8d9-2678-41e5-8633-c3942d8069e4	18582	Y	Y	Y	Y	Y	Y	Y	Y	Y
		N	N	N	N	N	N	N	N	N
be8f5123-872f-4dcb-ac96-2450e28041c1	19723	N	N	N	N	N	N	N	N	N
		N	N	N	N	N	N	N	N	N
40b10c52-6cba-45c4-af41-ed23081782b6	18428	Y	N	Y	Y	Y	Y	N	Y	Y
		N	N	Y	Y	Y	Y	N	Y	Y
03d4a4a9-e33a-4c53-9c55-b81aad73911e	19423	N	N	Y	Y	Y	Y	N	Y	Y
		N	N	Y	Y	Y	Y	N	Y	Y
0153c89e-aed5-48e9-a564-30fe5ceefb24	19357	Y	N	Y	Y	Y	Y	N	Y	Y
		N	N	N	N	N	N	N	N	N
9bba1062-9dd7-4d4a-a668-85b337fbbf64	18975	Y	Y	Y	Y	Y	Y	Y	Y	Y
		N	N	N	N	N	N	N	N	N
458066eb-478f-4814-aab1-cad1548c320e	19015	N	N	N	N	Y	Y	N	N	Y
		N	N	N	N	N	N	N	N	N
d1c8edef-fafc-4e98-8f9e-67ca5823243c	18756	Y	N	Y	Y	Y	N	N	Y	Y
		N	N	N	N	N	N	N	N	N
8987684c-859f-4ea7-9cd0-4371adc4a70e	18716	Y	N	Y	Y	Y	Y	N	Y	Y
		N	N	N	N	N	N	N	N	N
95733f11-aa48-4a2b-b478-cb02f4ded3ef	19085	Y	N	Y	Y	N	Y	Y	Y	Y
		N	N	N	N	N	N	N	N	N
61f986b2-a38e-4ce1-8c6d-4e32d4acebba	19275	Y	N	Y	Y	Y	Y	N	Y	Y
		N	N	Y	Y	Y	Y	N	Y	Y
7b7258d1-6569-42fd-94df-a41fdf462020	19628	Y	Y	Y	Y	Y	Y	Y	Y	Y
		N	Y	Y	Y	Y	Y	Y	Y	Y
a3f242b3-3e8c-40f7-9be0-7ff70e5d5690	18675	Y	Y	Y	Y	Y	Y	Y	Y	Y
		N	Y	Y	Y	Y	Y	Y	Y	Y
5bcbc416-f72a-46a0-a222-fb7b45758452	19691	Y	N	Y	Y	Y	Y	N	Y	Y
		N	N	Y	Y	Y	Y	N	Y	Y
136d3b26-a0a3-412c-99ec-ba3ece3e6177	17904	Y	Y	Y	Y	Y	Y	Y	Y	Y
		N	Y	Y	Y	Y	Y	Y	Y	Y
5be343d8-355e-446d-88bb-7f70574a583e	19849	N	N	Y	Y	Y	Y	N	Y	N
		N	N	N	N	N	N	N	N	N
f9860d61-19c4-4698-9557-b580a57e8e8c	19398	Y	N	Y	Y	Y	Y	N	Y	Y
		N	N	Y	Y	Y	Y	N	Y	Y
9517da5f-ff85-40c5-a348-1ce362a7b997	18863	Y	Y	Y	Y	Y	Y	Y	Y	Y
		N	N	Y	Y	Y	Y	N	Y	Y
